# Comparison of two crystal polymorphs of NowGFP reveals a new conformational state trapped by crystal packing

**DOI:** 10.1107/S2059798324008246

**Published:** 2024-09-02

**Authors:** Jin Kyun Kim, Hannah Jeong, Jeongwoo Seo, Seoyoon Kim, Kyung Hyun Kim, Duyoung Min, Chae Un Kim

**Affiliations:** a Department of Physics Ulsan National Institute of Science and Technology (UNIST) Ulsan44919 Republic of Korea; b Department of Chemistry Ulsan National Institute of Science and Technology (UNIST) Ulsan44919 Republic of Korea; c Department of Biotechnology and Bioinformatics Korea University Sejong30019 Republic of Korea; University of Queensland, Australia

**Keywords:** fluorescent proteins, NowGFP, crystal polymorphism, protein crystal packing, conformational flexibility, protein crystallization

## Abstract

Crystal polymorphism serves as a strategy to study the conformational flexibility of proteins. However, the relationship between protein crystal packing and protein conformation often remains elusive. In this study, two distinct crystal forms of a green fluorescent protein variant, NowGFP, are compared: a previously identified monoclinic form (space group *C*2) and a newly discovered ortho­rhombic form (space group *P*2_1_2_1_2_1_). Comparative analysis reveals that both crystal forms exhibit nearly identical linear assemblies of NowGFP molecules interconnected through similar crystal contacts. However, a notable difference lies in the stacking of these assemblies: parallel in the monoclinic form and perpendicular in the orthorhombic form. This distinct mode of stacking leads to different crystal contacts and induces structural alteration in one of the two molecules within the asymmetric unit of the orthorhombic crystal form. This new conformational state captured by orthorhombic crystal packing exhibits two unique features: a conformational shift of the β-barrel scaffold and a restriction of pH-dependent shifts of the key residue Lys61, which is crucial for the pH-dependent spectral shift of this protein. These findings demonstrate a clear connection between crystal packing and alternative conformational states of proteins, providing insights into how structural variations influence the function of fluorescent proteins.

## Abstract 2

Structures of NowGFP obtained from two distinct crystal forms were compared and a new conformational state captured by crystal packing was identified.

## 1. Introduction

Proteins can be crystallized in multiple forms, a phenomenon known as crystal polymorphism. A well-known example of protein crystal polymorphism is lysozyme, which exhibits six crystal polymorphs (Vaney *et al.*, 2001 ▸). The primary interest in discovering crystal polymorphs of proteins lies in finding a superior crystal form that enables the acquisition of detailed structural information through X-ray crystallography. This crystal form may either have high crystallinity for improved diffraction (Yamada *et al.*, 2017 ▸) or possess enhanced thermal and chemical durability (Gerlits *et al.*, 2019 ▸). Additionally, different crystal forms can reveal different conformation states of protein molecules which are trapped by crystal lattice packing (Jiang *et al.*, 2013 ▸). The availability of different crystal forms provides insight into the conformational flexibility of protein molecules and a detailed understanding of individual conformational states (Zhang *et al.*, 1995 ▸). In this study, we demonstrate a new case of protein crystal polymorphism with NowGFP, a variant of green fluorescent protein (GFP).

Fluorescent proteins have become essential tools in cell biology and biomedicine, serving as non-invasive methods for visualizing and tracking cellular and organism-wide processes (Chudakov *et al.*, 2010 ▸). Most of these fluorescent proteins are characterized by the tyrosine (Tyr) at the centre of the three residues responsible for forming the chromophore, resulting in fluorescence that spans from green to far-red wavelengths. Recently, a fluorescent protein based on an anionic tryptophan (Trp) was developed and was named WasCFP (Sarkisyan *et al.*, 2012 ▸). Derived from the cyan-fluorescent mCerulean (Rizzo *et al.*, 2004 ▸), it was engineered by introducing a critical Val61Lys substitution and four additional mutations. WasCFP was further improved to enhance its stability across a broad range of pH levels and temperatures, incorporating 13 additional mutations. This improved variant, which exhibits strong green fluorescence under physiological conditions, has been named NowGFP (Sarkisyan *et al.*, 2015 ▸).

Spectral and structural studies of NowGFP consistently reveal pH-dependent changes. Spectrally, NowGFP exhibits green fluorescence (λ_ex_/λ_em_ = 493/502 nm) at physiological and higher pH levels, contrasting with cyan fluorescence (λ_ex_/λ_em_ = 429/475 nm) under acidic conditions (pH < 6). The intensity ratio between green and cyan fluorescence is highly sensitive to pH conditions. Structurally, NowGFP adopts two distinct pH-dependent conformations at pH 4.8 and pH 9.0. These conformations involve the chromophore and the key residue Lys61, which is believed to play a central role in chromophore ionization and the resultant shifts in the fluorescence spectrum (Pletnev *et al.*, 2015 ▸).

We present three crystal structures of NowGFP obtained under various pH conditions from two distinct crystal forms: a monoclinic crystal structure at pH 4.8 (PDB entry 8xh0, 1.45 Å resolution) and orthorhombic crystal structures at pH 9.0 (PDB entry 8xh1, 1.7 Å resolution) and pH 6.0 (PDB entry 8xh2, 1.8 Å resolution). Our study begins with a detailed comparative analysis of crystal contacts and stacking inter­actions between these two crystal forms. Following this, we identify major differences in the crystal contacts across the two crystal forms and determine how the crystal packing may alter the NowGFP structure. Lastly, we investigate the alternative conformations trapped by the orthorhombic crystal form by examining five NowGFP molecules: one from the monoclinic form at pH 4.8, two from the orthorhombic form at pH 9.0 and two from the orthorhombic form at pH 6.0. This comparison aims to deepen our understanding of how crystal packing influences the pH-dependent conformational changes in NowGFP.

## 2. Materials and methods

### 2.1. Macromolecule production

The fragment encoding NowGFP with an N-terminal His tag and TEV protease recognition sequence site was cloned into the pET-24a(+) vector and transformed into *Escherichia coli* strain BL21(DE3) (Invitrogen, USA). Bacterial cultures were grown overnight at 16°C. Isopropyl β-d-1-thiogalactopyranoside (IPTG) induction was necessary for effective protein expression. The cells were pelleted by centrifugation, resuspended in phosphate-buffered saline (PBS) pH 7.4, 1 m*M* PMSF, 1 m*M* TCEP and lysed by an EmulsiFlex-C3 at 103–117 MPa. NowGFP was purified by immobilized metal-ion affinity chromatography using His60 Ni Superflow Resin (Clontech, USA) and then buffer-exchanged for cleavage by TEV protease on a Cytiva PD-10 DG column (1× TEV protease buffer: 25 m*M* Tris–HCl, 150 m*M* NaCl, 1 m*M* TCEP). TEV protease was applied (10 U µl^−1^, 1:100 ratio) and incubated at 30°C for 1 h. After incubation, further purification was achieved by size-exclusion chromatography using FPLC (ÄKTApure 25) with a HiLoad 16/600 Superdex 75 pg column (Cytiva, USA). The purity of the sample was then confirmed by SDS–PAGE analysis (Supplementary Fig. S1). The purified NowGFP protein was concentrated using an Amicon Ultra-15 centrifugal filter unit. The amino-acid sequence and other details are given in Table 1 ▸.

### 2.2. Crystallization

Crystallization screening was conducted to identify optimal conditions for the formation of NowGFP crystals. Single crystals in a monoclinic space group (*C*2) were obtained using KH_2_PO_4_ and PEG 3350. Additionally, single crystals in an ortho­rhombic space group (*P*2_1_2_1_2_1_) were obtained using sodium citrate and PEG 4000. Details of the crystallization screening, including other conditions that did not yield diffraction-quality single crystals, are provided in Supplementary Table S1.

To obtain monoclinic crystals, NowGFP was transferred to 20 m*M* Tris pH 8.0, 200 m*M* NaCl buffer and concentrated to 12 mg ml^−1^. Crystals suitable for data collection were obtained by the hanging-drop vapour-diffusion method (McPherson, 1982 ▸). Typically, 2 µl protein solution was mixed with an equal amount of reservoir solution and incubated at 4°C for a week. The best crystal was obtained from 16 m*M* KH_2_PO_4_ pH 4.8, 20%(*w*/*v*) PEG 3350.

To obtain orthorhombic crystals, NowGFP was transferred to 20 m*M* Tris pH 8.0, 200 m*M* NaCl buffer and concentrated to 17 mg ml^−1^. Crystals suitable for data collection were obtained by the hanging-drop vapour-diffusion method. Typically, 2 µl protein solution was mixed with an equal amount of reservoir solution and incubated at 4°C for a week. The best crystal was obtained from 100 m*M* sodium citrate pH 6.0, 25%(*w*/*v*) PEG 4000. To obtain structures of NowGFP at pH 9.0, the crystal was transferred to 100 m*M* ammonium sulfate, 50 m*M* Tris–HCl pH 9.0, 17.5%(*w*/*v*) PEG 4000 and incubated for a week. Details of protein crystallization and photographic images of crystals are given in Table 2 ▸ and Supplementary Fig. S2, respectively.

### 2.3. Data collection and processing

X-ray diffraction data were collected on Pohang Light Source-II (PLS-II) beamline 7A at the Pohang Accelerator Laboratory, Pohang, Republic of Korea. Prior to data collection, the monoclinic crystals were briefly soaked in a cryoprotectant solution consisting of 30%(*v*/*v*) glycerol and 70%(*v*/*v*) reservoir, while the orthorhombic crystals were soaked in a cryoprotectant solution consisting of 20%(*v*/*v*) glycerol and 80%(*v*/*v*) reservoir, and then flash-cooled in a 100 K nitrogen stream. A total of 360 images with 1° oscillation angles were collected with sample-to-detector distances of 150 and 175 mm for monoclinic and orthorhombic crystals, respectively. All diffraction images were processed with *HKL*-2000 (Otwinowski & Minor, 1997 ▸). The absorbed X-ray dose for a single data set was less than 5 × 10^5^ Gy, which is much lower than the Henderson dose limit of 1.45 × 10^7^ Gy (Henderson, 1990 ▸). Data-processing statistics and diffraction images are given in Table 3 ▸ and Supplementary Fig. S3, respectively.

### 2.4. Structure solution and model refinement

The structures of NowGFP from both crystal forms were determined using the *CCP*4 suite (Agirre *et al.*, 2023 ▸). The crystal structures were solved by the molecular-replacement method with *MOLREP* (Vagin & Teplyakov, 2010 ▸) using the previously solved structure of NowGFP (PDB entry 4rtc; Pletnev *et al.*, 2015 ▸) as a model. Crystallographic refinement was performed with *REFMAC*5 (Murshudov *et al.*, 2011 ▸), alternating with manual revision of the model with *Coot* (Emsley *et al.*, 2010 ▸). The location of water molecules and structure validation were performed with *Coot*. The monoclinic crystal structure (*C*2) of NowGFP at pH 4.8 was determined at 1.45 Å resolution and orthorhombic crystal structures (*P*2_1_2_1_2_1_) of NowGFP at pH 9.0 and 6.0 were solved at 1.7 and 1.8 Å resolution, respectively. The coordinates and structure factors for NowGFP in the monoclinic form at pH 4.8, the ortho­rhombic form at pH 9.0 and the orthorhombic form at pH 6.0 were deposited in the Protein Data Bank under accession codes 8xh0, 8xh1 and 8xh2, respectively. Structure-solution and data-refinement statistics are given in Table 4 ▸. All structural figures were rendered with *PyMOL* (Schrödinger).

## 3. Results and discussion

### 3.1. Crystal structures of two crystal polymorphs

**Figure 1:**
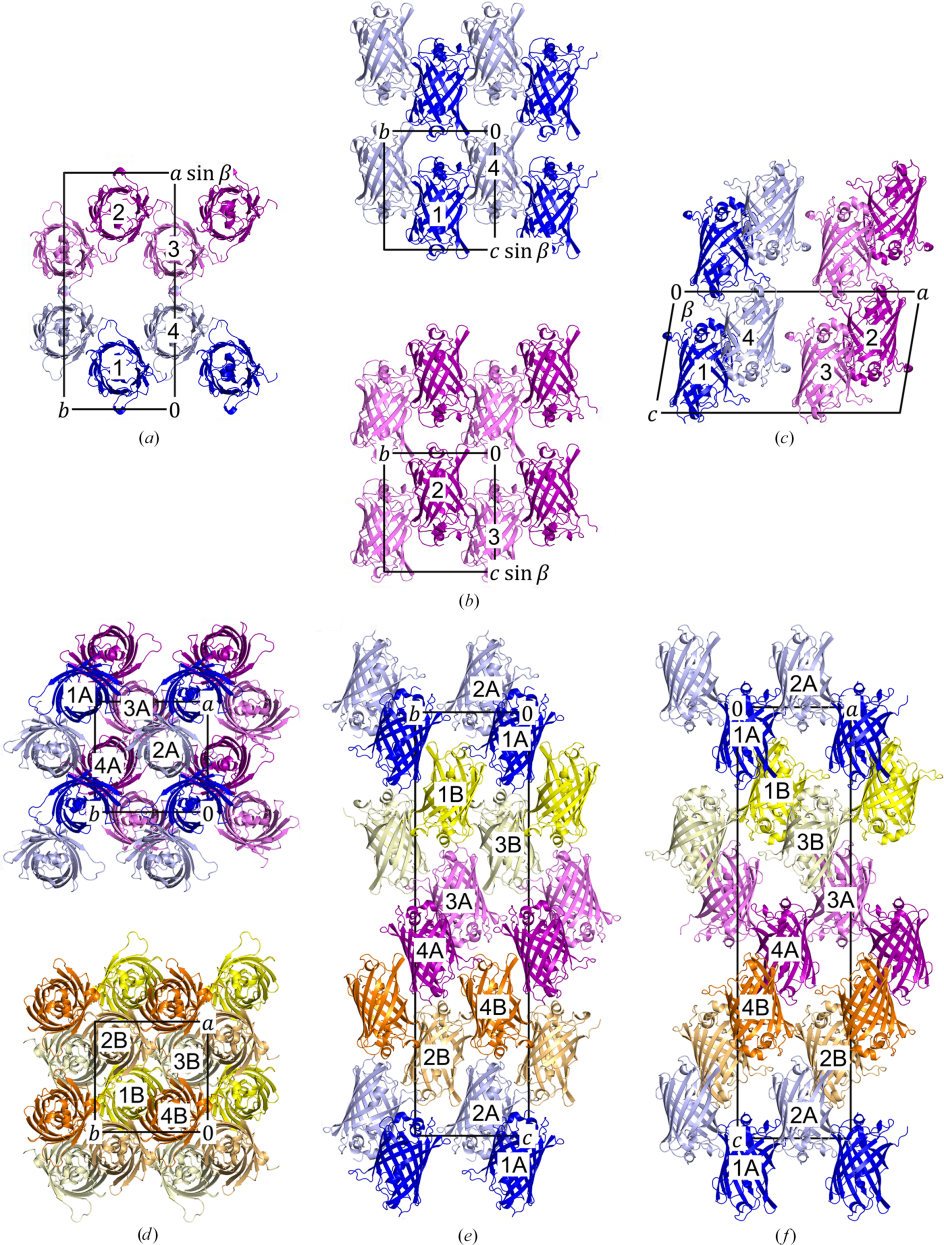
Assembly of the protein molecules in the monoclinic (*a*–*c*) and orthorhombic (*d*–*f*) crystal forms. Crystal structures are viewed along the *c* axis (*a*, *d*), *a* axis (*b*, *e*) and *b* axis (*c*, *f*). The secondary structures of NowGFP are represented as ribbons, while the unit cell of each crystal form is outlined in a black box. For the monoclinic form, molecules 1, 2, 3 and 4 are coloured blue, purple, light purple and light blue, respectively. For the orthorhombic form, molecules 1A, 2A, 3A, 4A, 1B, 2B, 3B and 4B are coloured blue, light blue, light purple, purple, yellow, light orange, light yellow and orange, respectively.

The assemblies of protein molecules within the unit cells of both orthorhombic and monoclinic crystals are illustrated in Fig. 1 ▸. For the comparative study of the two crystal forms, we chose the orthorhombic crystal structure obtained at pH 9.0 for comparison with the monoclinic structure since it has a higher resolution (1.7 Å). Comparison between the lower resolution orthorhombic crystal structure obtained at pH 6.0 (1.8 Å) and the monoclinic structure revealed nearly identical results to the higher pH (pH 9.0) counterpart. This comparison is detailed in Supplementary Figs. S4–S7 and Supplementary Tables S2 and S3.

In the case of the monoclinic (*C*2) crystal form there are four molecules inside the unit cell, and one molecule is in the asymmetric unit (chain *A*). We refer to four molecules, which are chains *A* from symmetry operations (*x*, *y*, *z*), (−*x*, *y*, −*z*), (*x* + 1/2, *y* + 1/2, *z*) and (−*x* + 1/2, *y* + 1/2, −*z*), as molecules 1, 2, 3 and 4, respectively (Figs. 1 ▸*a*–1 ▸*c*).

In the case of the orthorhombic (*P*2_1_2_1_2_1_) crystal form there are eight molecules inside the unit cell, and two molecules are in the asymmetric unit (chains *A* and *B*). We refer to four molecules, which are chains *A* from symmetry operations (*x*, *y*, *z*), (*x* + 1/2, −*y* + 1/2, −*z*), (−*x*, *y* + 1/2, −*z* + 1/2) and (−*x* + 1/2, −*y*, *z* + 1/2), as molecules 1A, 2A, 3A and 4A, respectively. Similarly, we refer to four molecules, which are chains *B* from symmetry operations (*x*, *y*, *z*), (*x* + 1/2, −*y* + 1/2, −*z*) (−*x*, *y* + 1/2, −*z* + 1/2) and (−*x* + 1/2, −*y*, *z* + 1/2) as molecules 1B, 2B, 3B and 4B, respectively (Figs. 1 ▸*d*–1 ▸*f*).

For simplicity, we refer to chain *A* of the NowGFP structure in the monoclinic form as Mono. Similarly, we refer to chains *A* and *B* in the orthorhombic form as Orth(A) and Orth(B), respectively. Comparative analysis of Mono, Orth(A) and Orth(B) indicates that there are no significant differences in the overall protein structures, with the C^α^–C^α^ r.m.s.d. being less than 0.4 Å. However, partial differences are observed in the r.m.s.d. from ideal angles, *B* factors and solvation free energy (Tables 4 ▸ and 5 ▸). These differences will be discussed in detail in Section 3.3.

### 3.2. Crystal contacts and related crystal packing

The term ‘crystal contacts’ denotes the interactions between protein molecules that emerge through the crystallization process (Janin & Rodier, 1995 ▸; Dasgupta *et al.*, 1997 ▸). In this section, the crystal contacts between NowGFP molecules are considered using *PISA* analysis (Krissinel & Henrick, 2005 ▸, 2007 ▸) and the protein crystal packing is discussed. All crystal contacts from each molecule are listed in Table 6 ▸.

**Figure 2:**
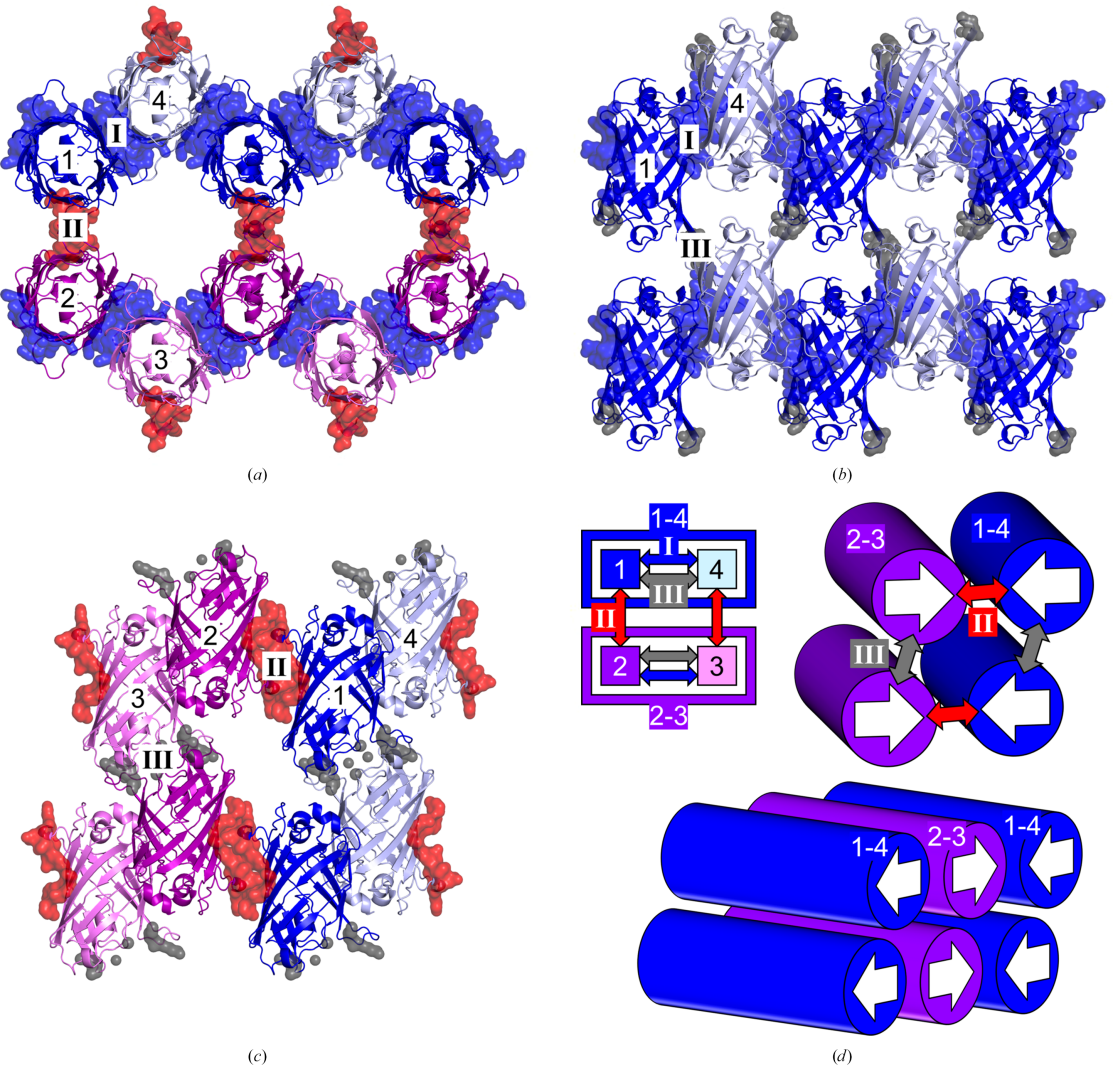
Crystal contacts and packing of NowGFP molecules in the monoclinic crystal structure. The secondary structures of NowGFP are represented as ribbons, and atoms belonging to crystal contacts are represented as surfaces. (*a*) Contacts I and II are viewed along the *c* axis. (*b*) Contacts I and III are viewed along the *a* axis. (*c*) Contacts II and III are viewed along the *b* axis. Contacts I, II and III are coloured blue, red and grey, respectively. (*d*) Schematic diagram of crystal packing. Crystal contacts are represented by coloured arrows, and linear assemblies connected by contact I are represented as blue- or purple-coloured boxes. Molecules connected by the blue-coloured contact I are linked along the *b* axis. Linear assemblies are stacked parallel by contacts II and III along the *a* axis and *c* axis, respectively. The symmetry operation between assemblies 1–4 and 2–3, indicated by opposite white arrows, is (−*x*, *y*, −*z*).

In the monoclinic (*C*2) crystal form, the crystal contacts are categorized into three types, denoted as contacts I, II and III. Contact I is between molecules 1 and 4 (or 2 and 3) (Figs. 2 ▸*a* and 2 ▸*b*), which has the largest contact area for Mono. More specifically, contact I comes from two different symmetry-related molecules (Mono) at (−*x* + 1/2, *y* − 1/2, −*z* − 1) and (−*x* + 1/2, *y* + 1/2, −*z* − 1) and connects molecules 1 and 4 (or 2 and 3) to form a zigzag linear assembly along the *b* axis (Fig. 2 ▸*d*). Contact II is between molecules 1 and 2 (or 3 and 4) (Figs. 2 ▸*a* and 2 ▸*c*). More specifically, contact II comes from symmetry-related molecules at (−*x*, *y*, −*z* − 1) and connects two types of linear assemblies (1–4 and 2–3) to form layers in the *ab* plane (Fig. 2 ▸*d*). The symmetry operation between two neighbouring linear assemblies is (−*x*, *y*, −*z*), which is the same as the symmetry operation between molecules 1 and 2 or 3 and 4 (represented as opposite white arrows in Fig. 2 ▸*d*). Contact III is between molecules 1 and 3 (or 2 and 4), which has the smallest contact area (Figs. 2 ▸*a* and 2 ▸*b*). More specifically, contact III comes from two different symmetry-related molecules at (−*x* + 1/2, *y* − 1/2, −*z*) and (−*x* + 1/2, *y* + 1/2, −*z*) and results from the packing of sheets along the *c* axis (Fig. 2 ▸*d*).

**Figure 3:**
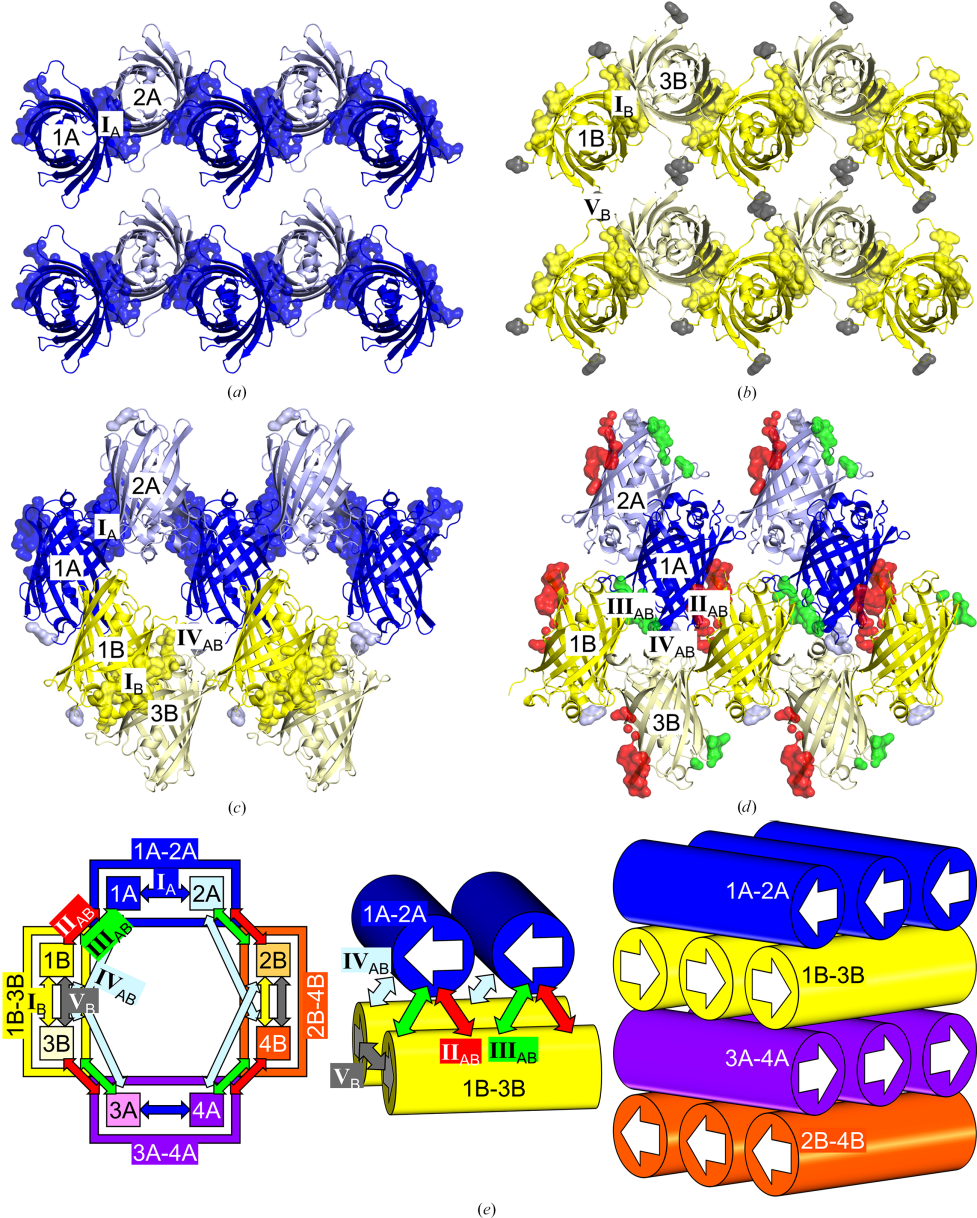
Crystal contacts and packing of NowGFP molecules in the orthorhombic crystal structure. The secondary structures of NowGFP are represented as ribbons, and atoms belonging to crystal contacts are represented as surfaces. (*a*) Contact I_A_ is viewed along the *c* axis. (*b*) Contacts I_B_ and V_B_ are viewed along the *c* axis. (*c*) Contacts I_A_ and I_B_ are viewed along the *b* axis. (*d*) Contacts II_AB_, III_AB_ and IV_AB_ are viewed along the *a* axis. Contacts I_A_, I_B_, II_AB_, III_AB_, IV_AB_ and V_B_ are coloured blue, yellow, red, green, light blue and grey, respectively. (*e*) Schematic diagram of protein packing. Crystal contacts are represented by coloured arrows and linear assemblies connected by contact I_A_ are represented as blue- or purple-coloured boxes, while linear assemblies connected by contact I_B_ are represented as yellow- or orange-coloured boxes. The linear assemblies connected by contacts I_A_ and I_B_ are linked along the *a* axis and the *b* axis, respectively. Linear assemblies composed of chains *A* and *B* are stacked perpendicular by contacts II_AB_, III_AB_ and IV_AB_ along the *c* axis. Linear assemblies composed of chain *B* are stacked parallel by contact V_B_ along the *a* axis. The symmetry operation between two assemblies composed of the same chains, 1A–2A and 3A–4A (or 1B–3B and 2B–4B), indicated by opposite white arrows, is (−*x* + 1/2, −*y*, *z* + 1/2).

In the orthorhombic (*P*2_1_2_1_2_1_) crystal form, the crystal contacts are categorized into six types: contacts I_A_, I_B_, II_AB_, III_AB_, IV_AB_ and V_B_. Contact I_A_ is between molecules 1A and 2A (or 3A and 4A) (Figs. 3 ▸*a* and 3 ▸*c*), which has the largest contact area for Orth(A). More specifically, contact I_A_ comes from symmetry-related molecules chain *A* [Orth(A)] at (*x* − 1/2, −*y* + 1/2, −*z*) and (*x* + 1/2, −*y* + 1/2, −*z*) and connects molecules 1A and 2A (or 3A and 4A) to form a zigzag linear assembly along the *a* axis (Fig. 3 ▸*e*). Contact I_B_ is between molecules 1B and 3B (or 2B and 4B) (Figs. 3 ▸*b* and 3 ▸*c*), which has the largest contact area for Orth(B). More specifically, contact I_B_ comes from symmetry-related molecules chain *B* [Orth (B)] at (−*x* + 1, *y* − 1/2, −*z* + 1/2) and (−*x* + 1, *y* + 1/2, −*z* + 1/2) to form a zigzag linear assembly along the *b* axis (Fig. 3 ▸*e*). Contacts II_AB_ and III_AB_ are between molecules 1A and 1B (or 2A and 2B, 3A and 3B, or 4A and 4B) (Fig. 3 ▸*d*). More specifically, contact II_AB_ comes from chain *A* and chain *B* in the same asymmetric unit, while contact III_AB_ comes from a symmetry-related chain *B* (*x*, *y* + 1, *z*) for chain *A* (*x*, *y*, *z*). Contact IV_AB_ is between molecules 1A and 3B (or 2A and 4B, 3A and 1B, or 4A and 2B) (Fig. 3 ▸*d*). More specifically, contact IV_AB_ comes from a symmetry-related chain *B* (−*x*, *y* + 1/2, −*z* + 1/2) for chain *A* (*x*, *y*, *z*). Contacts II_AB_, III_AB_ and IV_AB_ connect two types of linear assemblies which are orthogonal to each other (assembly of molecules 1A–2A and 1B–3B, 1A–2A and 2B–4B, 3A–4A and 1B–3B, or 3A–4A and 2B–4B; Fig. 3 ▸*e*), and II_AB_ has the largest contact area among them. The symmetry operation between two linear assemblies made of the same chains (chain *A* or *B*) is (−*x* + 1/2, −*y*, *z* + 1/2). This is the same as the symmetry operation between molecules 1A and 4A (or 1B or 4B, 2A and 3A, or 2B and 3B) and is represented as opposite white arrows in Fig. 3 ▸(*e*). Contact V_B_ is between molecules 1B and 3B (or 2B and 4B), which has the smallest contact area (Fig. 3 ▸*b*). More specifically, contact V_B_ comes from a symmetry-related chain *B* at (−*x*, *y* − 1/2, −*z* + 1/2) and (−*x*, *y* + 1/2, −*z* + 1/2).

### 3.3. The structural alteration of NowGFP results from crystal contacts

**Figure 4:**
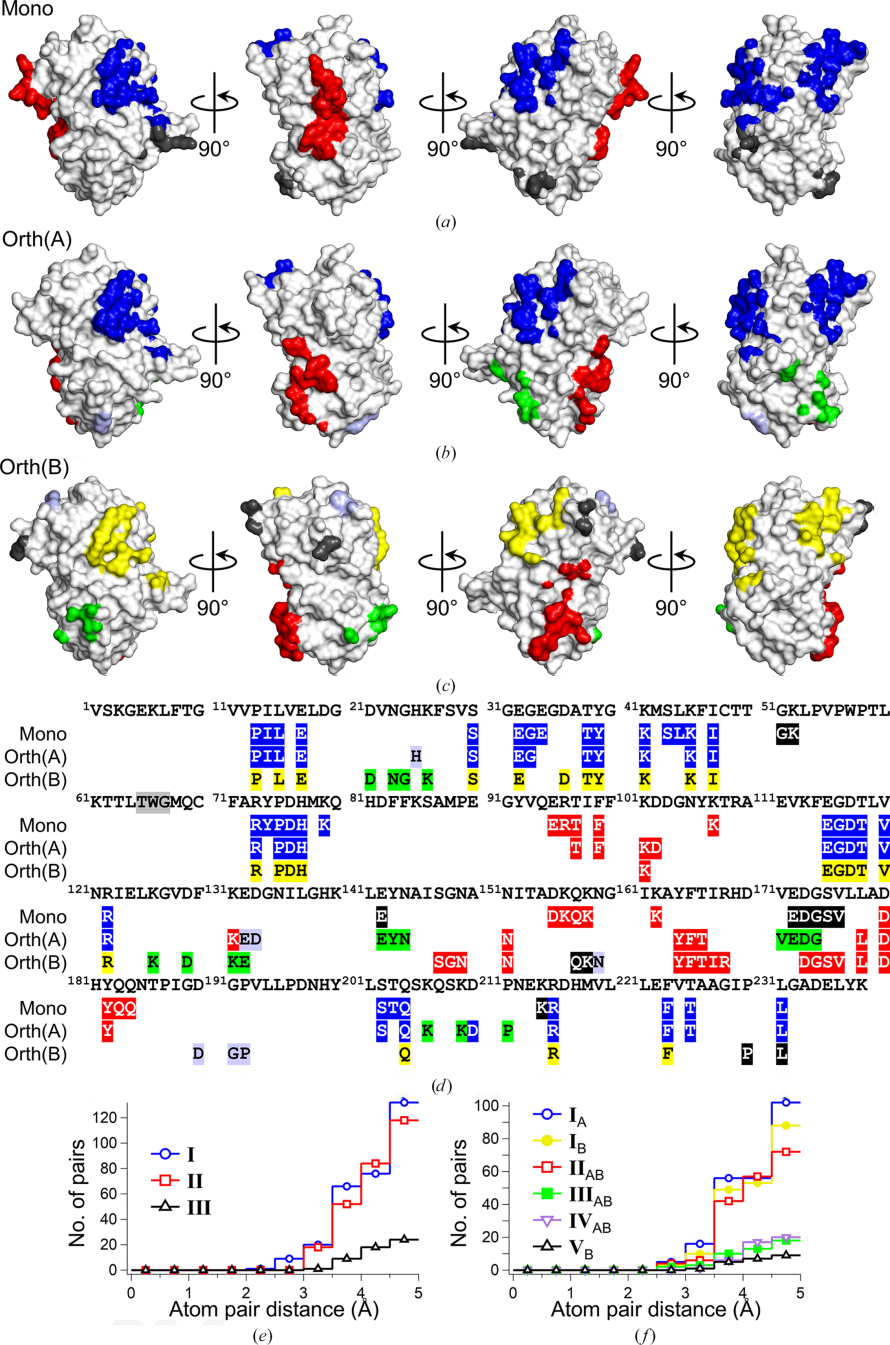
Comparison of crystal contacts for three types of molecules. (*a*) Chain *A* of the monoclinic structure, Mono. 1, 2, 3 and 4 are identical. (*b*) Chain *A* of the orthorhombic structure, Orth(A). 1A, 2A, 3A and 4A are identical. (*c*) Chain *B* of the orthorhombic structure, Orth(B). 1B, 2B, 3B and 4B are identical. (*d*) Amino-acid sequence representation of the three molecules. Thr65, Trp66 and Gly67 were replaced by the chromophore. For Mono, atoms belonging to contacts I, II and III are coloured blue, red and grey, respectively. For Orth(A) and Orth(B), atoms belonging to contacts I_A_, I_B_, II_AB_, III_AB_, IV_AB_ and V_B_ are coloured blue, yellow, red, green, light blue and grey, respectively. Histograms of the distances between atom pairs in each crystal contact from (*e*) the monoclinic and (*f*) the orthorhombic crystal forms are shown.

**Figure 5:**
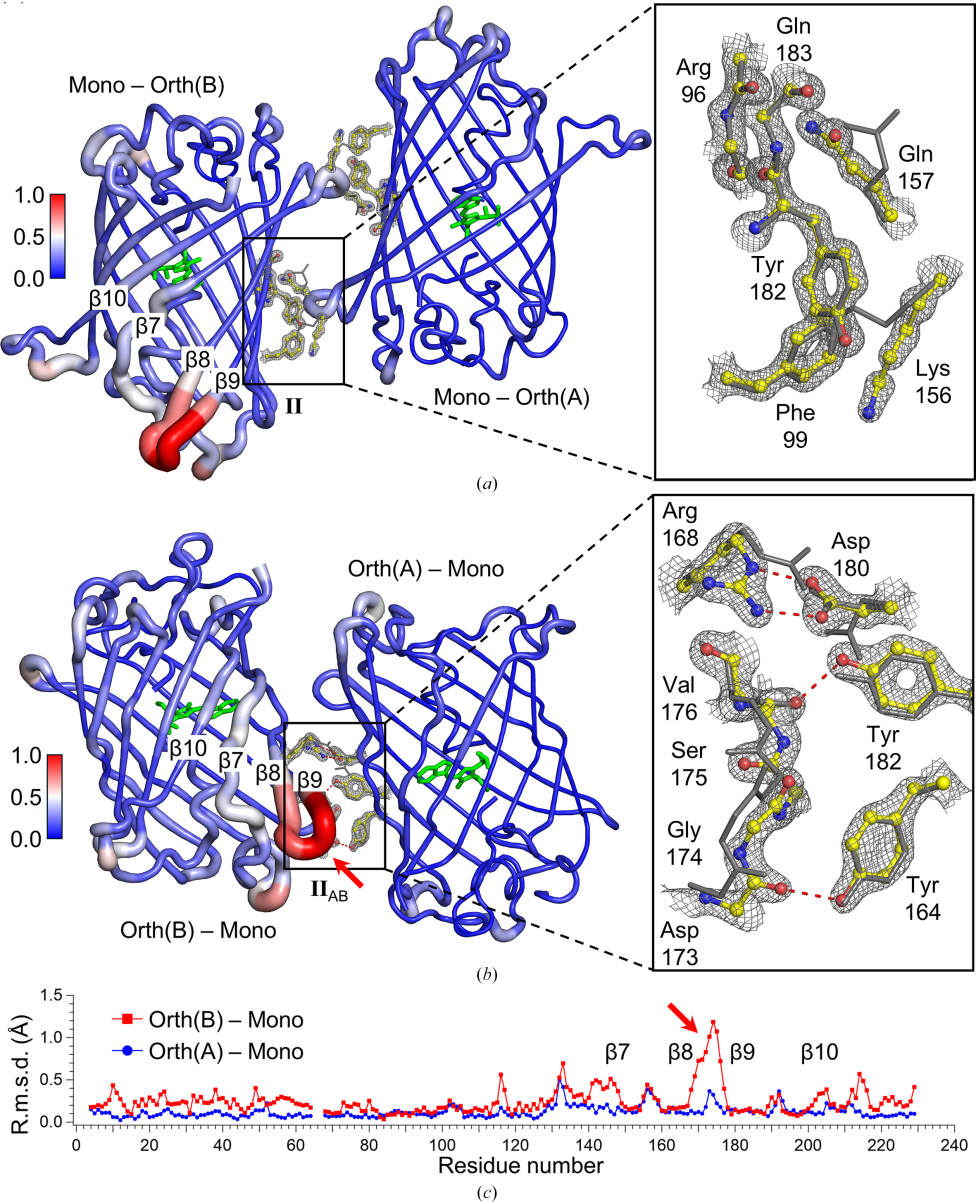
Comparison of contacts II and II_AB_. (*a*) Two molecules in the monoclinic crystal structure connected by contact II are presented. The Orth(A) and Orth(B) structures are superposed with the Mono structure and coloured grey. For clarity, only the main chains of Arg96 and Gln183 are represented. (*b*) Two molecules from the orthorhombic structure connected by contact II_AB_ are presented. The Mono structures are superposed with Orth(A) and Orth(B) and coloured grey. For clarity, only the main chains of Val176, Ser175, Gly174 and Asp173 are represented. The r.m.s.d. between the main chains of two superposed molecules is shown as a putty representation, with a blue–white–red colour profile. Crystal contacts less than 3.0 Å are represented as red dotted lines. Stick representations of chromophores are coloured green. The electron-density map (2*F*_o_ − *F*_c_ map) has a cutoff level of 2.0σ. (*c*) The r.m.s.d. plot of the main-chain atoms of two structures from different crystal forms. Residues close to the N-terminus and C-terminus are ignored, and only the residues numbered from 4 to 229 are considered. Residue numbers 65, 66 and 67 are ignored since these are substituted with the chromophore. The region with the most significant r.m.s.d., spanning residues 170–176, is indicated by a red arrow.

Comparison of the crystal contacts of three types of molecules [Mono, Orth(A) and Orth(B)] from two different crystal forms shows that contacts I, I_A_ and I_B_ are similar in structure and sequence (blue and yellow in Fig. 4 ▸). Notably, linear assemblies derived from contacts I, I_A_ and I_B_ are also almost identical (Figs. 2 ▸*a*, 2 ▸*b* and 3 ▸*a*–3 ▸*c*). However, there is a significant difference between contacts II and II_AB_ (red in Fig. 4 ▸). This difference is responsible for the packing mode of the linear assemblies: parallel for the monoclinic form and perpendicular for the orthorhombic form. There are minor differences in the other contacts, including contacts III, III_AB_, IV_AB_ and V_B_, but those have a much smaller contact area compared with contacts I, I_A_, I_B_, II and II_AB_ (Table 6 ▸). In addition, the histogram of the distances between atom pairs for each crystal contact indicates that contacts I, I_A_, I_B_, II and II_AB_ are more closely interacting compared with others (Figs. 4 ▸*e* and 4 ▸*f*). We thus focused on analysing the differences between contacts II and II_AB_ as represented in Fig. 5 ▸.

For contact II in the monoclinic crystal form, there are no atom pairs closer than 3.0 Å (Table 6 ▸). Phe99, Tyr182, Gln157 and Lys156 from two molecules contact each other without any water molecule in between. Gln157 also contacts the main chain of Arg96 and Gln183. Additionally, residues corresponding to contact II show no significant deviation from Mono to Orth(A) and Orth(B) (Fig. 5 ▸*a*). This indicates that contact II does not significantly influence the overall protein structure.

For contact II_AB_ in the orthorhombic crystal form, there are four atom pairs closer than 3.0 Å (Table 6 ▸). Two of them come from the arginine–aspartic acid salt bridge between Asp180 of Orth(A) and Arg168 of Orth(B). The other two are from Tyr182 and Tyr164 of Orth(A), which are connected to the main chain of Val176 and Asp173 of Orth(B) through hydrogen bonding. Compared with Mono, a significant shift of the main chain is observed for residues 170–176 of Orth(B), which mainly involves contact II_AB_ and the loop between the β8 and β9 strands (Figs. 5 ▸*b* and 5 ▸*c*). This indicates that contact II_AB_ might be responsible for the structural shifts in Orth(B). Additionally, this shift causes residues 141–147, which are part of the β7 strand, to move away from the β10 strand (Fig. 5 ▸). Specifically, the C^α^–C^α^ distances between Asn144 in the β7 strand and Gln207 in the β10 strand are 6.3, 6.1 and 6.7 Å for Mono, Orth(A) and Orth(B), respectively.

The evidence that Orth(B) has a more unstable structure compared with Orth(A) or Mono is that the r.m.s.d. from ideal angles, average *B* factor and solvation free energy all show the highest values for Orth(B). In detail, the r.m.s.d.s from ideal angles for Mono, Orth(A) and Orth(B) are ∼2.0°, ∼1.9° and ∼2.1°, respectively, the average *B* factors of the main chain from Mono, Orth(A) and Orth(B) are ∼22.7, ∼26.8 and 31.0 Å^2^, respectively, and the solvation free energy from the isolated structure of each molecule is ∼−215 kcal mol^−1^ for Orth(A) and Mono and ∼−209 kcal mol^−1^ for Orth(B) (Tables 4 ▸ and 5 ▸). These results indicate that Orth(A) exhibits similar conformational behaviour to Mono, while Orth(B) represents a relatively unstable conformational state.

Moreover, protein crystals are highly hydrated, with those with lower solvent content typically having better diffraction quality (Matthews, 1968 ▸; Zhang *et al.*, 1995 ▸). However, solvent-content estimation reveals that the orthorhombic crystal has less solvent (∼50%) yet results in a lower resolution (1.7 Å) compared with the monoclinic crystal, which contains more solvent (∼58%) but diffracts to a higher resolution (1.45 Å) (Table 3 ▸). This discrepancy could be additional evidence that the crystallinity of the orthorhombic crystal is poorer due to the instability of Orth(B).

### 3.4. New conformational state of NowGFP trapped by crystal lattice packing

In a previous study by Pletnev and coworkers, it was revealed that the key residue Lys61 plays a central role in the ionization process of the chromophore, showing significant pH-dependent conformational changes. Specifically, at high pH the N^ζ^ atom of Lys61 makes two hydrogen bonds: one to the N^ɛ^ atom of Trp66 and the other to the O^ɛ1^ atom of Glu222 (*k*1 conformation). This dual bonding helps to form a non­’;covalent connection and promotes the deprotonation of Trp66. In contrast, at lower pH the orientation of Lys61 shifts away from Trp66, instead forming a hydrogen bond to the N^ɛ2^ atom of Gln207 (*k*2 conformation; Pletnev *et al.*, 2015 ▸).

To elucidate the alternative conformation state trapped by the orthorhombic crystal lattice, a total of five NowGFP molecules are compared (one molecule from the monoclinic crystal structure at pH 4.8 and four molecules from the orthorhombic crystal structures at pH 6.0 and pH 9.0).

**Figure 6:**
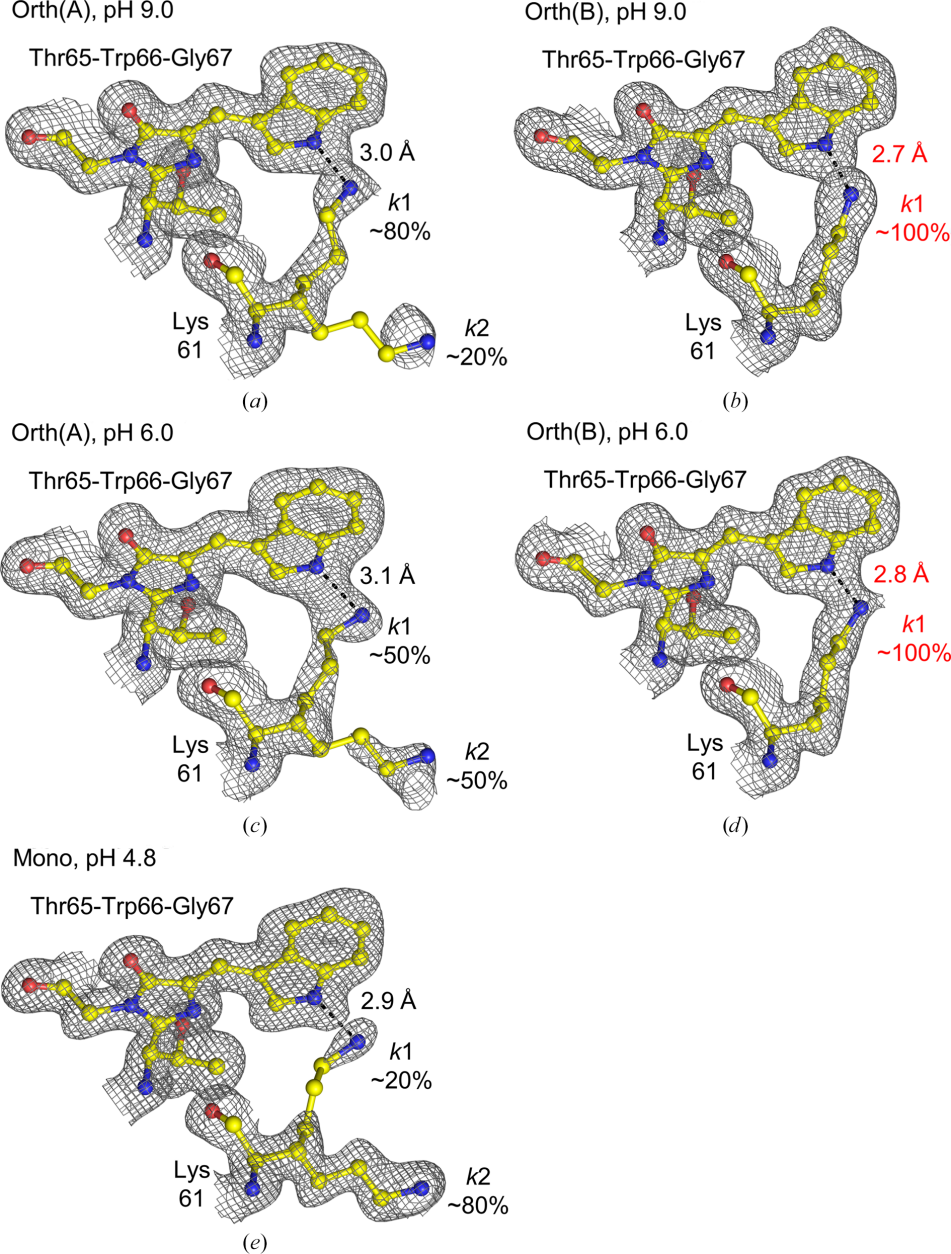
The chromophore Thr65-Trp66-Gly67 and key residue Lys61 under various pH conditions. The structures are from (*a*, *b*) the orthorhombic crystal at pH 9.0, (*c*, *d*) the orthorhombic crystal at pH 6.0 and (*e*) the monoclinic crystal at pH 4.8. The electron-density map (2*F*_o_ − *F*_c_ map) has a cutoff level of 0.8σ. Black dotted lines between the N^ɛ^ atom of the Trp66 indole and the N^ζ^ atom of Lys61 represent hydrogen bonding. Orth(B), which is a new conformational state of NowGFP caused by an orthorhombic crystal lattice, shows the dominant *k*1 conformation of Lys61, while both Mono and Orth(A) exhibit pH-dependent conformational shifts of Lys61.

In the case of the Orth(A) and Mono structures, the key residue Lys61 has ∼80% *k*1 conformation and ∼20% *k*2 conformation under a high-pH condition (pH 9.0). This shifts to ∼50% *k*1 conformation and ∼50% *k*2 conformation as the pH decreases to 6.0, and finally to ∼20% *k*1 conformation and ∼80% *k*2 conformation under an acidic pH condition (pH 4.8) (Figs. 6 ▸*a*, 6 ▸*c* and 6 ▸*e*). This trend of conformational shifts from *k*1 to *k*2 as the pH decreases is consistent with previously published structures: the monoclinic structure at pH 9.0 (PDB entry 4rtc, 1.18 Å resolution) and pH 4.8 (PDB entry 4rys, 1.35 Å resolution) (Supplementary Fig. S8; Pletnev *et al.*, 2015 ▸). Even though the high-resolution structure of NowGFP shows a partial *trans* conformation of the chromophore at pH 4.8, this is difficult to observe in our moderate-resolution structure (1.45 Å).

In the case of the Orth(B) structure, however, the conformation of Lys61 is almost 100% *k*1 at both pH 9.0 and pH 6.0, and the distance between the N^ɛ^ atoms of the Trp66 indole and the N^ζ^ atom of Lys61 in the *k*1 conformation is ∼0.3 Å closer compared with the Orth(A) structure (Figs. 6 ▸*a*–6 ▸*d*). It appears that strong hydrogen bonding between the Trp66 indole and the *k*1 conformation of Lys61 is responsible for the high occupancies of the *k*1 conformation in the Orth(B) structure. Considering that Orth(B) has an unstable structure compared with Orth(A) and Mono, and this instability is linked to crystal contact II_AB_ as detailed in Section 3.3, it is evident that the alteration of the Lys61 conformation in the Orth(B) structure is a result of orthorhombic crystal packing. Table 7 ▸ shows the χ_1_ and χ_2_ angles of two alternative conformations of the key residue Lys61.

While this alternative conformational state could be dismissed as merely an artefact of crystal packing, a previous study of crystal polymorphs of photoactive yellow protein indicates that the crystal lattice does not simply impose arbitrary conformational changes on the protein molecule (van Aalten *et al.*, 2000 ▸). Instead, the protein shifts along essential eigenvectors to adapt to different lattice environments. Therefore, different protein structures from crystal polymorphs may represent the inherent conformational flexibility of the molecule (van Aalten *et al.*, 1997 ▸).

Previous studies, including the unfolding and backbone dynamics of GFP, reveal that β-strands 7, 8, 9 and 10 exhibit greater flexibility (Huang *et al.*, 2007 ▸; Seifert *et al.*, 2003 ▸). Additionally, the parental WasCFP shows conformational flexibility in the β7 and β10 strands, which opens the solvent channel between these two β-strands. This flexibility is expected to play a crucial role in managing solvent access to the chromophore (Laricheva *et al.*, 2015 ▸). These observations are consistent with our finding that the r.m.s.d. of the main-chain atoms between two structures from crystal polymorphs shows higher values at residues corresponding to β-strands 7, 8, 9 and 10. The most significant deviations are noted in the loop between the β8 and β9 strands, resulting in opening of the solvent channel between the β7 and β10 strands (Fig. 5 ▸). Based on these correspondences, the new conformational state of NowGFP captured by crystal packing in this study is likely to represent one of the potential conformational states inherent to this protein.

## 4. Conclusions

In this study, we report the discovery of a novel orthorhombic crystal form of NowGFP and conduct a detailed comparison with the known monoclinic crystal form. Our investigations primarily focused on the crystal contacts, revealing that both forms exhibit similar zigzag linear assemblies of protein molecules resulting from crystal contact I. The key distinction between the two forms lies in their stacking modes: parallel stacking for the monoclinic form and perpendicular stacking for the orthorhombic form. This difference in packing correlates with a specific crystal contact, referred to as crystal contact II (or II_AB_), and results in an alteration of one molecule in the symmetry unit of the orthorhombic crystal form, designated as Orth(B). Given that these structural shifts are predominantly concentrated between β-strands 7–10, which are known for their partial flexibility, we propose that this altered molecule represents an alternative conformational state of NowGFP. In contrast, the other molecule in the orthorhombic form, Orth(A), remains unchanged and is similar to that found in the monoclinic form.

Significantly, this new conformational state of NowGFP captured in the orthorhombic crystal packing exhibits a different functional behaviour: the key residue Lys61, which is known for its pH-dependent shift from the *k*1 to the *k*2 conformation, appears to be locked in the *k*1 configuration regardless of pH conditions. This contrasts with the unaltered molecule, in which Lys61 exhibits pH-dependent movement as expected. These observations provide valuable insights into how crystal lattice packing influences the conformational states of protein molecules, enhancing our understanding of the conformational flexibility of protein structures.

## Supplementary Material

Supplementary figures and tables. DOI: 10.1107/S2059798324008246/jb5066sup1.pdf

PDB reference: NowGFP, monoclinic form obtained at pH 4.8, 8xh0

PDB reference: orthorhombic form obtained at pH 9.0, 8xh1

PDB reference: orthorhombic form obtained at pH 6.0, 8xh2

## Figures and Tables

**Table 1:** Macromolecule-production information for NowGFP

DNA source	Synthetic DNA
Forward primer	T7 promotor
Reverse primer	T7 terminator
Expression vector	pET-24a(+)
Expression host	*E. coli* BL21(DE3)
Complete amino-acid sequence of the construct produced	SVSKGEKLFTGVVPILVELDGDVNGHKFSVSGEGEGDATYGKMSLKFICTTGKLPVPWPTLKTTLTWGMQCFARYPDHMKQHDFFKSAMPEGYVQERTIFFKDDGNYKTRAEVKFEGDTLVNRIELKGVDFKEDGNILGHKLEYNAISGNANITADKQKNGIKAYFTIRHDVEDGSVLLADHYQQNTPIGDGPVLLPDNHYLSTQSKQSKDPNEKRDHMVLLEFVTAAGIPLGADELYK

**Table 2:** Crystallization conditions for the two crystal forms of NowGFP

Structure	Monoclinic pH 4.8	Orthorhombic pH 9.0	Orthorhombic pH 6.0
PDB code	8xh0	8xh1	8xh2
Method	Hanging drop	Hanging drop	Hanging drop
Plate type	24-well protein-crystallization plate	24-well protein-crystallization plate	24-well protein-crystallization plate
Temperature (K)	277	277	277
Protein concentration (mg ml^−1^)	12.1	17.4	17.4
Buffer composition of protein solution	20 m*M* Tris pH 8.0, 200 m*M* NaCl	20 m*M* Tris pH 8.0, 200 m*M* NaCl	20 m*M* Tris pH 8.0, 200 m*M* NaCl
Composition of reservoir solution	16 m*M* KH_2_PO_4_ pH 4.8, 20%(*w*/*v*) PEG 3350	100 m*M* sodium citrate pH 6.0, 25%(*w*/*v*) PEG 4000	100 m*M* sodium citrate pH 6.0, 25%(*w*/*v*) PEG 4000
Composition of soaking solution†	—	100 m*M* ammonium sulfate, 50 m*M* Tris–HCl pH 9.0, 17.5%(*w*/*v*) PEG 4000	—
Volume and ratio of drop	2 µl:2 µl	2 µl:2 µl	2 µl:2 µl
Volume of reservoir (µl)	500	500	500

† The soaking solution is used to adjust crystals to different pH conditions.

**Table 3:** Data-collection statistics for NowGFP crystals Values in parentheses are for the outer shell.

Structure	Monoclinic pH 4.8	Orthorhombic pH 9.0	Orthorhombic pH 6.0
PDB code	8xh0	8xh1	8xh2
Diffraction source	PLS-II 7A	PLS-II 7A	PLS-II 7A
Wavelength (Å)	0.97934	0.97934	0.97934
Temperature (K)	100	100	100
Detector	ADSC Quantum 270	ADSC Quantum 270	ADSC Quantum 270
Crystal-to-detector distance (mm)	150	175	175
Rotation range per image (°)	1	1	1
Total rotation range (°)	360	360	360
Exposure time per image (s)	1	1	1
Space group	*C*2	*P*2_1_2_1_2_1_	*P*2_1_2_1_2_1_
*a*, *b*, *c* (Å)	110.55, 51.04, 55.52	51.24, 52.28, 195.30	51.17, 51.71, 196.24
α, β, γ (°)	90, 99.58, 90	90, 90, 90	90, 90, 90
*Z*/*Z*′	4/1	8/2	8/2
Estimated solvent content (%)	57.5	49.9	51.3
Resolution range (Å)	30–1.45	30–1.70	30–1.80
Total No. of reflections	401360	846596	709367
No. of unique reflections	54859	59042	49354
Completeness (%)	99.8	99.9	100.0
Multiplicity	7.3	14.3	14.4
〈*I*/σ(*I*)〉	37.3 (2.5)	36.7 (3.5)	39.6 (3.6)
*R*_r.i.m._	0.072	0.079	0.071

**Table 4:** Structure-refinement statistics for NowGFP crystals Values in parentheses are for the outer shell.

Structure	Monoclinic pH 4.8	Orthorhombic pH 9.0	Orthorhombic pH 6.0
PDB code	8xh0	8xh1	8xh2
Resolution	30.0–1.45 (1.48–1.45)	30.0–1.70 (1.73–1.70)	30.0–1.80 (1.83–1.80)
Completeness (%)	100.0	100.0	99.5
No. of reflections, working set	51316	55947	46765
No. of reflections, test set	2809	2870	2489
Final *R*_cryst_	0.127	0.153	0.149
Final *R*_free_	0.158	0.220	0.229
No. of non-H atoms			
Chain *A*	1893	1827	1818
Chain *B*	—	1809	1809
Glycerol	2	2	2
Water	266	229	159
R.m.s. deviations of bond lengths (Å)			
Chain *A*	0.0165	0.0158	0.0163
Chain *B*	—	0.0162	0.0164
R.m.s. deviations of angles (°)			
Chain *A*	2.01	1.92	2.02
Chain *B*	—	2.10	2.09
Average *B* factors (Å^2^)			
Chain *A* (main/side chain)	22.69/25.30	26.75/33.02	27.00/33.26
Chain *B* (main/side chain)	—	30.98/37.49	29.47/36.52
Glycerol	37.92	55.35	42.38
Water	38.50	33.63	33.13
Ramachandran statistics (%)			
Preferred	97.76	98.43	97.76
Allowed	2.24	1.57	2.24
Outliers	—	—	—

**Table 5:** Protein molecules from the monoclinic crystal form and the orthorhombic crystal form The total numbers of atoms and residues in the refined NowGFP molecule (residues 2–231) are 1809 and 228, respectively. H atoms are not considered. All values are from *PISA* analysis (Krissinel & Henrick, 2005 ▸, 2007 ▸).

Crystal form	Name of molecule	No. of atoms on the surface†	No. of residues on the surface†	Surface area‡ (Å^2^)	Solvation energy‡ (kcal mol^−1^)
Monoclinic	Mono	1022	210	10614.4	–215.1
Orthorhombic	Orth(A)	998	211	10620.8	–214.9
Orthorhombic	Orth(B)	993	210	10438.4	–209.2

† Solvent-accessible surface area of the corresponding structure.

‡ Solvation energy gain upon protein folding.

**Table 6:** Crystal contacts of the monoclinic crystal form and the orthorhombic crystal form Symmetry operations for symmetry-related neighbouring molecules are given in parentheses. H atoms are not considered. All values are from *PISA* analysis (Krissinel & Henrick, 2005, 2007 ▸).

Contacting molecule	Name of contact	No. of atoms in the contact	No. of residues in the contact	Contact area† (Å^2^)	Solvation energy‡ (kcal mol^−1^)	No. of atom pairs closer than 3.0/3.5 Å
Mono						
(−*x* + 1/2, *y* − 1/2, −*z* − 1)	I	52	15	525.3	−2.0	10/30
(−*x* + 1/2, *y* + 1/2, −*z* − 1)	I	61	19	502.8	−1.0	10/30
(−*x*, *y*, −*z* − 1)	II	61	14	512.3	1.2	0/18
(−*x* + 1/2, *y* − 1/2, −*z*)	III	13	6	123.0	−0.4	0/1
(−*x* + 1/2, *y* + 1/2, −*z*)	III	11	4	119.6	−1.0	0/1
Orth(A)						
*A* (*x* − 1/2, −*y* + 1/2, −*z*)	I_A_	52	17	492.9	0.7	5/21
*A* (*x* + 1/2, −*y* + 1/2, −*z*)	I_A_	50	12	475.8	−1.2	5/21
*B* (*x*, *y*, *z*)	II_AB_	50	12	404.6	0.0	4/10
*B* (*x*, *y* + 1, *z*)	III_AB_	26	10	186.4	−0.2	2/5
*B* (−*x*, *y* + 1/2, −*z* + 1/2)	IV_AB_	9	3	76.6	−0.7	0/1
Orth(B)						
*B* (−*x* + 1, *y* − 1/2, −*z* + 1/2)	I_B_	45	14	422.1	−0.6	3/13
*B* (−*x* + 1, *y* + 1/2, −*z* + 1/2)	I_B_	41	10	438.0	0.1	3/13
*A* (*x*, *y*, *z*)	II_AB_	45	16	411.0	−1.1	4/10
*A* (*x*, *y* − 1, *z*)	III_AB_	21	8	183.2	0.5	2/5
*A* (−*x*, *y* − 1/2, −*z* + 1/2)	IV_AB_	9	4	76.1	–0.6	0/1
*B* (−*x*, *y* − 1/2, −*z* + 1/2)	V_B_	8	2	77.3	0.1	0/1
*B* (−*x*, *y* + 1/2, −*z* + 1/2)	V_B_	6	2	76.5	–1.2	0/1

† Surface area buried by the intermolecular interface.

‡ Solvation energy gain upon formation of the interface. This value does not include the effect of satisfied hydrogen bonds and salt bridges across the interface.

**Table 7:** Two alternative conformations of the key residue Lys61

Name of molecule	pH	χ_1_/χ_2_ of *k*1 conformation (°)†	χ_1_/χ_2_ of *k*2 conformation (°)†
Orth(A) pH 9.0	9.0	−158/−152 (∼80%)	−74/−175 (∼20%)
Orth(B) pH 9.0	9.0	−169/−103 (∼100%)	—
Orth(A) pH 6.0	6.0	−166/–132 (∼50%)	−84/−179 (∼50%)
Orth(B) pH 6.0	6.0	−165/−101 (∼100%)	—
Mono pH 4.8	4.8	−169/–103 (∼20%)	–72/−181 (∼80%)

† χ_1_/χ_2_ are the torsion angles corresponding to C^α^—C^β^/C^β^—C^γ^. The partial occupancies of the alternative conformational states are given in parentheses.
