# Supplementary material for: Comparison of two crystal polymorphs of NowGFP reveals a new conformational state trapped by crystal packing
Source: Acta Crystallogr D Struct Biol. 2024 Sep 2;80(Pt 9):686–98. doi: 10.1107/S2059798324008246 (PMC11394120; doi:10.1107/S2059798324008246)
Supplement: Supplementary file 1 [file d-80-00686-sup1.pdf]

STRUCTURAL
BIOLOGY

Volume 80 (2024)

Supporting information for article:

Comparison of two crystal polymorphs of NowGFP reveals a new conformational state trapped by crystal packing

Jin Kyun Kim, Hannah Jeong, Jeongwoo Seo, Seoyoon Kim, Kyung Hyun Kim, Duyoung Min and Chae Un Kim

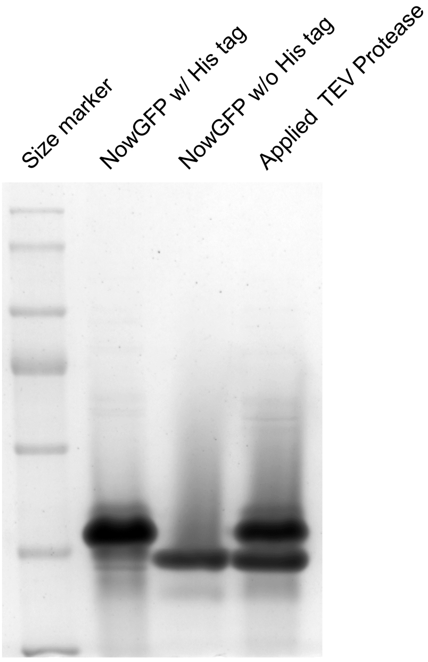

Figure S1 The 8 % SDS-PAGE gel image of NowGFP with His tag, NowGFP without His tag, and the applied TEV Protease. NowGFP without His tag was used for the crystallization. Details can be found in Table 1.

Table S1 Screening of crystallization conditions of NowGFP.

Salt	Precipitant	pH	Temperature	Crystal forms
100 mM Sodium citrate	5–25 % PEG 4000	4.8	4 ℃	–
100 mM Sodium citrate	5–25 % PEG 4000	5.0	4 ℃	–
100 mM Sodium citrate	5–25 % PEG 4000	5.5	4 ℃	–
100 mM Sodium citrate	5–30 % PEG 4000	6.0	4 ℃	Orthorhombic
100 mM Sodium acetate	5–25 % PEG 4000	9.0	4 ℃	–
100 mM Ammonium sulphate	5–30 % PEG 5000 monomethyl	4.5	20 ℃	–
10–20 mM KH_2PO_4	10–24 % PEG 3350	4.8	20 ℃	Monoclinic
10–20 mM KH_2PO_4	10–18 % PEG 3350	5.2	20 ℃	–

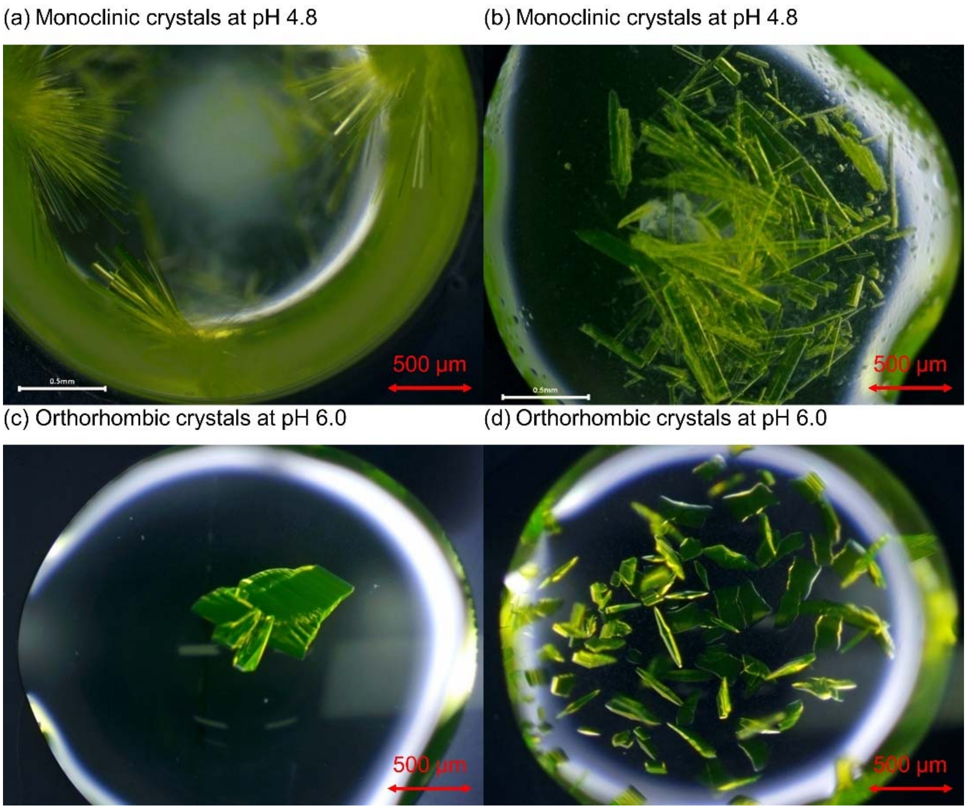

Figure S2 Photographic view of the NowGFP crystals: (a-b) the monoclinic and (c-d) the orthorhombic crystal forms. (a) Needle-shaped crystals grew along the b-axis of the monoclinic unit cell. After several tries, the best quality crystals, shown in (b), were selected for the X-ray diffraction data collection. (c-d) The orthorhombic crystals typically exhibited a plate-shaped morphology. The best quality crystals, shown in (d), were selected for the X-ray diffraction data collection. For the orthorhombic crystal structure obtained at pH 9.0, crystals initially grown at pH 6.0 were used, followed by an adjustment of the pH values through soaking in a pH 9.0 solution. All crystals were grown using the hanging drop vapor diffusion method. Details can be found in Table 2.

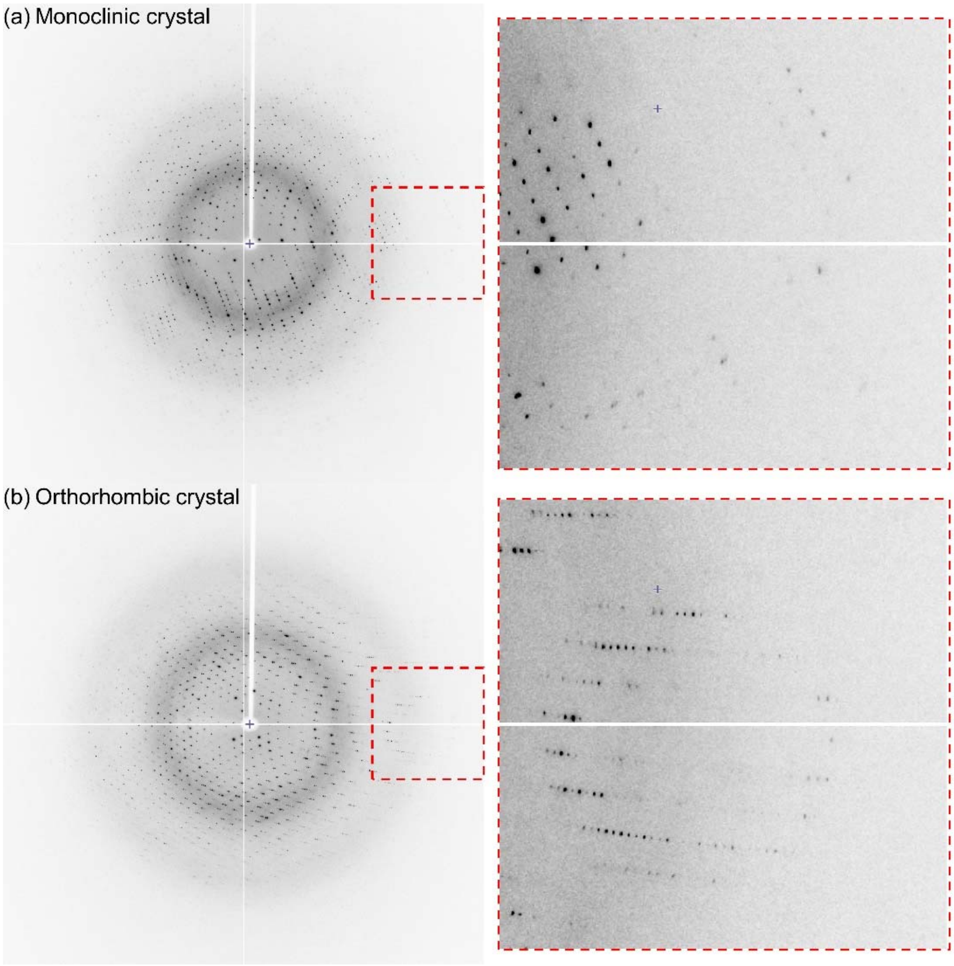

Figure S3 Representative diffraction images of two NowGFP crystal forms. (a) The monoclinic form at pH 4.8 results in 1.45 Å resolution. (b) The orthorhombic form at pH 9.0 results in 1.7 Å resolution. Total 360 diffraction images were collected for each crystal from PLS II 7A beamline with 1° oscillation angle and 0.97934 Å of wavelength. The space group belongs to *C*2 for the monoclinic form and $P2_12_12_1$ for the orthorhombic form, respectively. Details can be found in Table 3.

Table S2 Protein molecules from the orthorhombic crystal form under pH 6.0 condition

Crystal form	Name of molecule	No. of atoms on the surface	No. of residues on the surface	Surface area † (Å^2)	Solvation energy ‡ (kcal/mol)
Orthorhombic	Orth(A)	1004	211	10618.5	–215.5
Orthorhombic	Orth(B)	991	211	10463.5	–211.2

* The total numbers of atoms and residues in the refined NowGFP molecule (residues from 2 to 231) are 1809 and 228, respectively. Hydrogen atoms are not considered. All values are from PISA analysis (Krissinel & Henrick, 2005, Krissinel & Henrick, 2007).

† Solvent-accessible surface area of the corresponding structure

‡ Solvation energy gain upon protein folding.

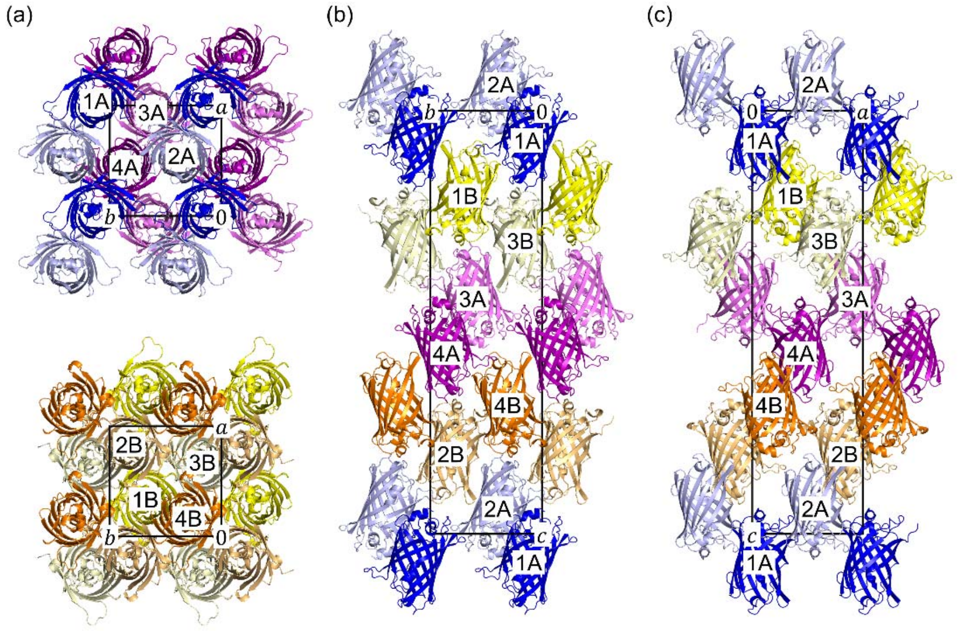

Figure S4 Assembly of the protein molecules inside the orthorhombic crystal forms at pH 6.0. Crystal structures are viewed along the c-axis (a), a-axis (b), and b-axis (c), respectively. The secondary structures of NowGFP are represented as ribbons, while the unit cell of each crystal form is outlined in a black box. Molecules 1A, 2A, 3A, 4A, 1B, 2B, 3B, and 4B are coloured blue, light blue, light purple, purple, yellow, light orange, light yellow, and orange, respectively. Note that the pH value does not affect the overall orthorhombic crystal packing.

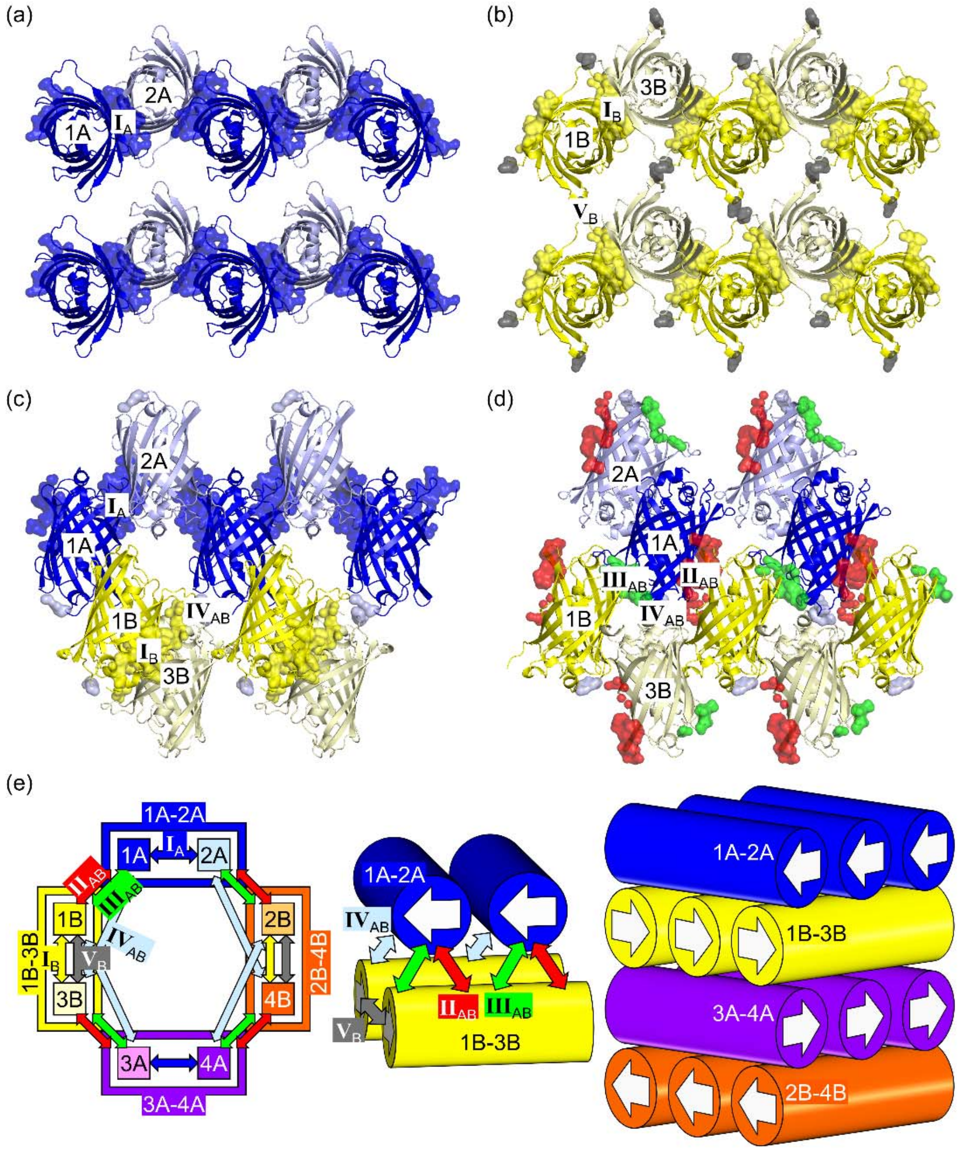

Figure S5 Crystal contacts and packing of NowGFP molecules from the orthorhombic crystal structure at pH 6.0. The secondary structures of NowGFP are represented as ribbons, and atoms belonging to crystal contact are represented as surfaces. (a) Contact I_A is viewed along the c-axis (b) Contact I_B and V_B are viewed along the c-axis. (c) Contacts I_A and I_B are viewed along b-axis. (d) Contacts II_{AB}, III_{AB}, and IV_{AB} are viewed along the a-axis. Contacts I_A, I_B, II_{AB}, III_{AB}, IV_{AB}, and V_B are coloured in blue, yellow, red, green, light blue, and grey respectively. (e) The schematic diagram of protein packing. Crystal contacts are represented coloured arrows and linear assemblies connected by contact I_A are represented as blue or purple-coloured box, while linear assemblies connected by contact I_B are represented as yellow or orange-coloured box. The linear assemblies connected by contacts I_A and I_B are linked along the a-axis and the b-axis, respectively. Each linear assemblies composed of chain A and B are stacked perpendicular by contact II_{AB}, III_{AB}, and IV_{AB} along the c-axis. Linear assemblies composed of chain B are stacked parallel by contact V_B along the a-axis. The

symmetry operation between two assemblies composed of the same chains 1A-2A and 3A-4A (or 1B-3B and 2B-4B), indicated by opposite white arrows, is (−x+1/2, −y, z+1/2). Note that the pH value does not affect the overall orthorhombic crystal packing.

Table S3 Crystal contacts of the orthorhombic crystal form under pH 6.0 condition

Orth(A)						
Contacting molecule	Name of contact	No. of atoms on the contact	No. of residues on the contact	Contact area † (Å^2)	Solvation energy gain ‡ (kcal/mol)	No. of atom pairs closer than 3.0/3.5 Å
A (x−1/2, −y+1/2, −z)	I_A	52	18	498.9	0.1	5/25
A (x+1/2, −y+1/2, −z)	I_A	51	14	485.9	–2.0	5/25
B (x, y, z)	II_{AB}	50	12	403.5	0.1	4/6
B (x, y+1, z)	III_{AB}	29	11	204.4	–0.4	2/9
B (−x, y+1/2, −z+1/2)	IV_{AB}	10	3	93.1	–0.9	0/1
Orth(B)						
Contacting molecule	Name of contact	No. of atoms on the contact	No. of residues on the contact	Contact area † (Å^2)	Solvation energy gain ‡ (kcal/mol)	No. of atom pairs closer than 3.0/3.5 Å
B (−x+1, y−1/2, −z+1/2)	I_B	47	15	424.2	0.1	5/20
B (−x+1, y+1/2, −z+1/2)	I_B	42	12	444.8	0.5	5/20
A (x, y, z)	II_{AB}	46	16	408.3	–1.0	4/6
A (x, y−1, z)	III_{AB}	23	8	207.5	0.4	2/9
A (−x, y−1/2, −z+1/2)	IV_{AB}	13	4	89.5	–0.7	0/1
B (−x, y−1/2, −z+1/2)	V_B	6	1	64.2	0.3	0/2
B (−x, y+1/2, −z+1/2)	V_B	6	2	61.8	–1.0	0/2

* Values in parentheses are symmetry operations for this symmetry-related neighbouring molecules. Hydrogen atoms are not considered. All values are from PISA analysis (Krissinel & Henrick, 2005, Krissinel & Henrick, 2007).

† Surface area buried by the inter-molecular interface.

‡ Solvation energy gain upon formation of the interface. This value does not include the effect of satisfied hydrogen bonds and salt bridges across the interface.

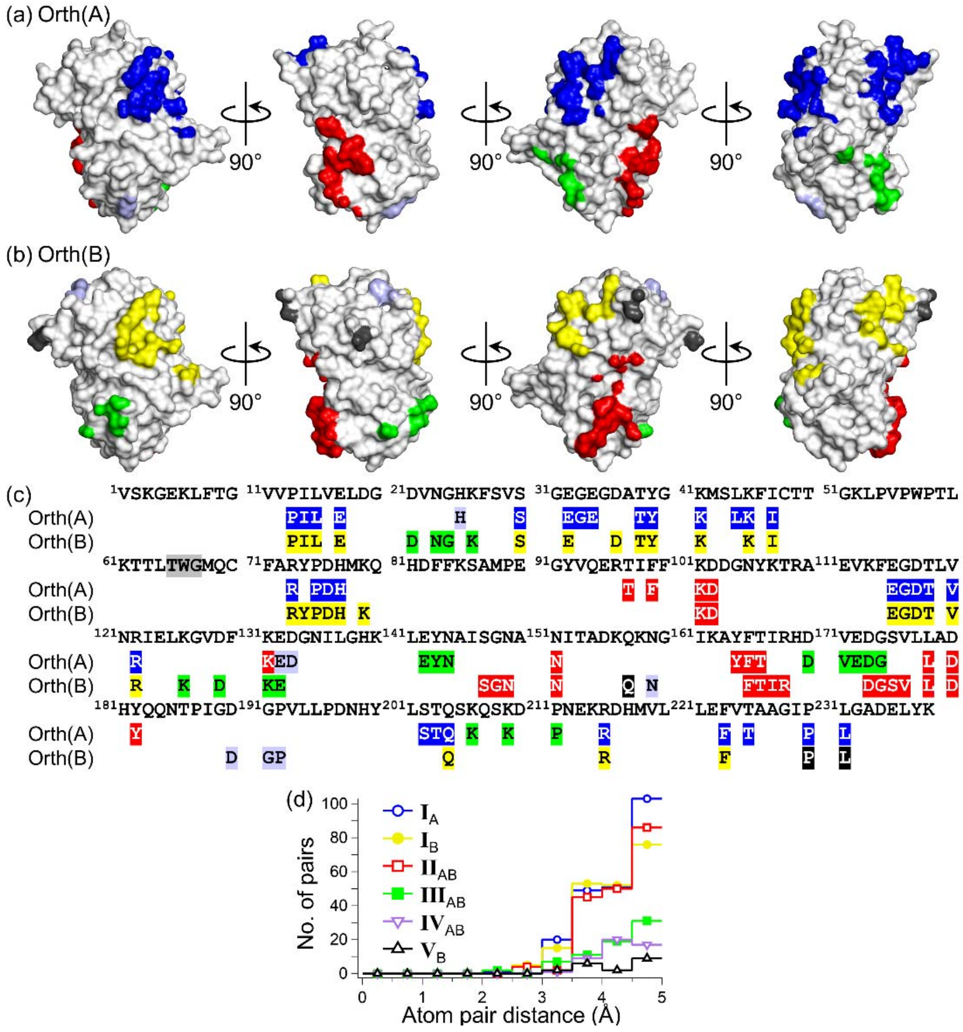

Figure S6 Comparison of crystal contacts for two types of molecules. (a) Chain A of the orthorhombic structure at pH 6.0. All 1A, 2A, 3A, and 4A are identical. (b) Chain B of the orthorhombic structure at pH 6.0. All 1B, 2B, 3B, and 4B are identical. (c) Amino acid sequence representation for the two molecules. Thr65, Trp66, and Gly67 were replaced by chromophore. Atoms belonging to contacts I_A, I_B, II_{AB}, III_{AB}, IV_{AB}, and V_B are coloured in blue, yellow, red, green, light blue, and grey respectively. (d) The histogram of the distances between atom pairs in each crystal contact from the orthorhombic crystal form at pH 6.0. Note that the pH value does not affect the crystal contact of the orthorhombic crystal form.

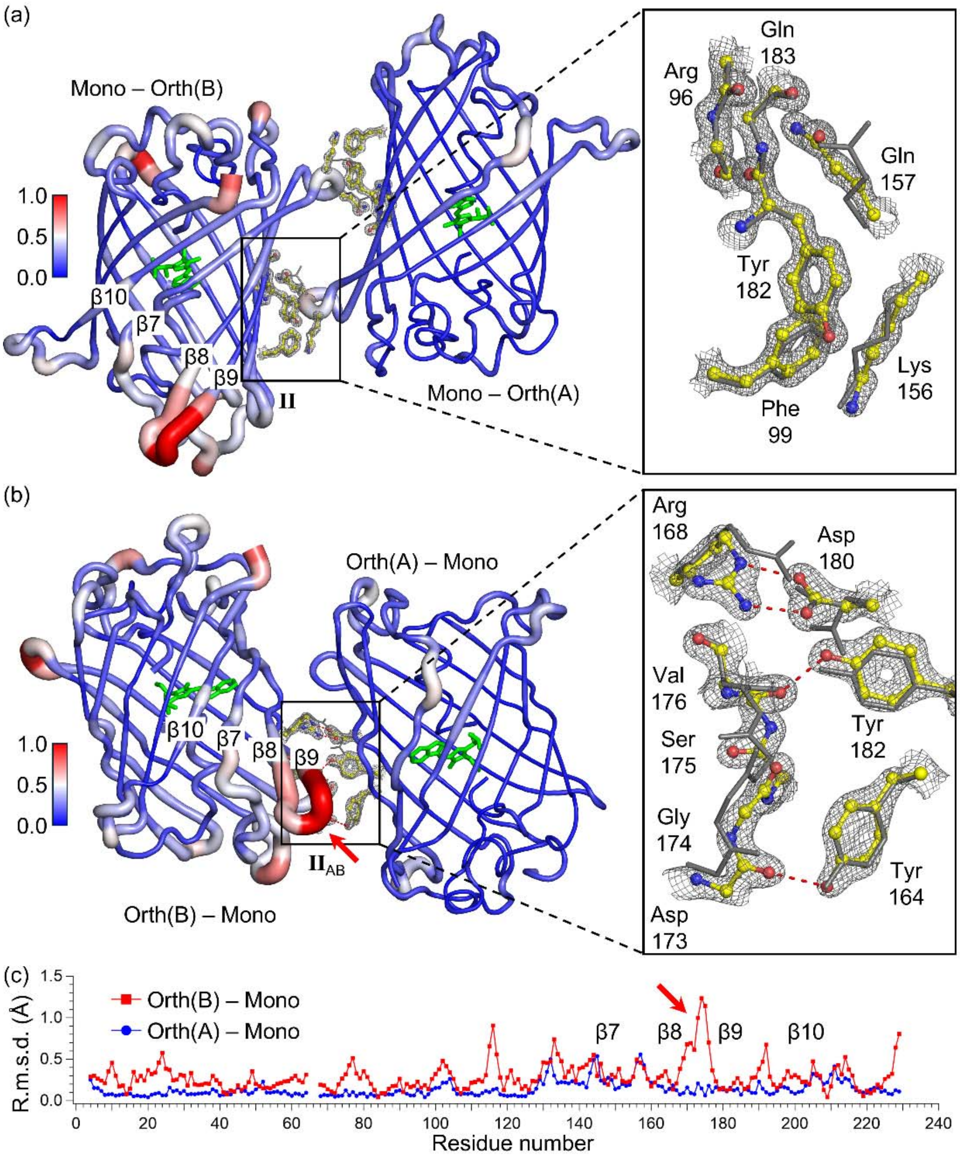

Figure S7 Comparison of contact II and II_{AB}. (a) Two molecules from the monoclinic crystal structure connected by contact II are presented. The Orth(A) and Orth(B) structures at pH 6.0 are superposed with Mono structure and coloured grey. For clarity, only the main chains of Arg96 and Gln183 are represented. (b) Two molecules from the orthorhombic structure connected by contact II_{AB} are presented. The Mono structures are superposed with Orth(A) and Orth(B) at pH 6.0 and coloured grey. For clarity, only the main chains of Val176, Ser175, Gly174, and Asp173 are represented. The r.m.s.d. between the main chains of two superposed molecules is shown as a putty representation, with a blue-white-red colour profile. Crystal contacts less than 3.0 Å are represented as red dotted lines. Stick representations of chromophores are coloured as green. The electron density map ($2F_o{-}F_c$ map) has a cutoff level of 2.0σ. (c) The r.m.s.d. plot of the main chain atoms of two structures from different crystal forms. Residues close to the N-terminal and C-terminal are ignored, and only the residues numbered from 4 to 229 are considered. Residue number 65, 66, and 67 are ignored since

those are substituted with chromophore. The region with the most significant r.m.s.d., spanning from residues 170 to 176, is indicated by a red arrow. Note that the pH value does not affect the crystal contact of the orthorhombic crystal form.

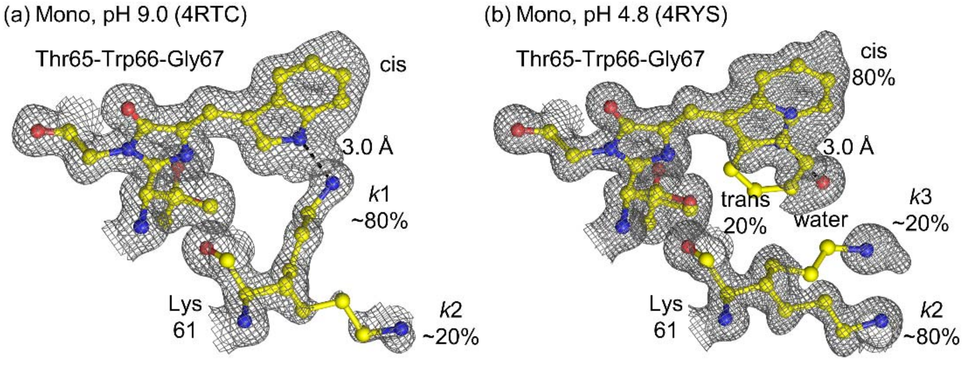

Figure S8 The chromophore Thr65-Trp66-Gly67 and key residue Lys61 from various pH conditions (Pletnev *et al.*,2015). Each structure is from (a) the monoclinic crystal under pH 9.0 condition and (b) the monoclinic crystal under pH 4.8 condition. The electron density map ($2F_o{-}F_c$ map) has a cutoff level of 0.8σ. Black dotted lines between the N_ε atom of Trp66 indole and the N_ζ atom of Lys61 represent hydrogen bonding.
